# A phase 1 randomized safety, reactogenicity, and immunogenicity study of Typhax: A novel protein capsular matrix vaccine candidate for the prevention of typhoid fever

**DOI:** 10.1371/journal.pntd.0007912

**Published:** 2020-01-06

**Authors:** Robert T. Cartee, Ann Thanawastien, Thomas J. Griffin IV, John J. Mekalanos, Stephan Bart, Kevin P. Killeen

**Affiliations:** 1 Matrivax Research & Development Corporation, Boston, MA, United States of America; 2 Department of Microbiology, Harvard Medical School, Boston MA, United States of America; 3 Trial Professionals Consulting Group Inc., Woodstock, MD, United States of America; Massachusetts General Hospital, UNITED STATES

## Abstract

**Background:**

Typhoid fever remains a significant cause of morbidity and mortality in developing countries especially in children ≤5 years old. Although the widely available unconjugated Vi polysaccharide vaccines are efficacious, they confer limited, short-term protection and are not approved for young children or infants. Vi conjugate vaccines, however, are now licensed in several typhoid endemic countries for use in children >6 months of age. As an alternative to conjugate vaccines, Matrivax has applied its novel ‘virtual conjugation’ Protein Capsular Matrix Vaccine (PCMV) technology to manufacture Typhax, which is composed of Vi polysaccharide entrapped in a cross-linked CRM197 matrix.

**Methodology:**

A randomized, double-blinded, dose escalating Phase 1 study was performed to compare the safety and immunogenicity of three dose levels of aluminum phosphate adjuvanted Typhax (0.5, 2.5, or 10 μg of Vi antigen) to the FDA licensed vaccine, Typhim Vi, and placebo. Groups of 15 healthy adult subjects aged 18 to 55 years were randomized and received Typhax, Typhim Vi, or placebo at a ratio of 9:3:3. Typhax and placebo were administered in a two-dose regimen (Days 0 and 28) while Typhim Vi was administered as a single-dose on Day 0 with a placebo administered on Day 28. All doses were administered as a 0.5 mL intramuscular (IM) injection in a blinded fashion. The anti-Vi IgG antibody response was determined preimmunization (Day 0) and on Days 14, 28, 42, and 180 by ELISA. Seroconversion was defined as a titer 4-fold or greater above baseline.

**Principal findings:**

All Typhax vaccine regimens were well tolerated and adverse events were low in number and primarily characterized as mild in intensity and similar in incidence across the treatment groups. Reactogenicity, primarily pain and tenderness at the injection site, was observed in both the Typhax and Typhim Vi treatment groups; a modest increase in incidence was observed with increasing Typhax doses. Following one dose of Typhax, seroconversion rates at day 28 were 12.5%, 77.8%, 66.7% at the 0.5, 2.5, and 10 μg dose levels, respectively, compared to 55.6% and 0% in the Typhim Vi and placebo groups, respectively. A second dose of Typhax on Day 28 did not elicit a significant increase in GMT or seroconversion at Day 42 or Day 180 at any dose level.

**Conclusions:**

Collectively, the results from this randomized phase 1 clinical trial indicate that Typhax is safe, well tolerated, and immunogenic. After a single dose, Typhax at the 2.5 and 10 μg dose levels elicited comparable anti-Vi IgG titers and seroconversion rates as a single dose of Typhim Vi (25 μg dose). A second dose of Typhax at Day 28 did not elicit a booster response.

**Trial registration:**

ClinicalTrials.gov NCT03926455.

## Author summary

Typhoid fever is a disease caused by the bacterium *Salmonella enterica* serovar Typhi and remains a significant health burden in the developing world, with an estimated 11 million cases that result in ~116,000 annual deaths. Although the widely available unconjugated typhoid fever vaccines provide some level of protection for adults, they are not approved for young children and infants, whom are at higher risk of disease than previously documented. However, conjugate vaccines that target the predominant cell surface Vi polysaccharide have been recently approved for use in these two age groups in endemic typhoid areas. As an alternative to conjugate vaccines, Matrivax has developed Typhax, a novel typhoid Protein Capsular Matrix Vaccine, or ‘virtual conjugate’ that consists of Vi antigen, non-covalently entrapped in a protein matrix. Here we describe the safety and immunogenicity results of a Phase 1 clinical study of three dose levels of Typhax administered in a two-dose regimen in healthy subjects. Overall, Typhax was well tolerated at all three dose levels and elicited comparable antibody titers and seroconversion at the 2.5 and 10 μg dose levels to the commercial FDA licensed vaccine, Typhim Vi (25 μg dose) after a single administration. These findings support the further clinical evaluation of Typhax as a typhoid fever vaccine candidate.

## Introduction

Typhoid fever, caused by *Salmonella enterica* serovar Typhi (*Salmonella* Typhi), remains a significant cause of morbidity and mortality particularly in tropical regions of the world with a recent 2017 study estimating 11 million cases per year that resulted in ~116,000 deaths [1]. A recent surveillance study in sub-Saharan Africa showed the incidence rate of typhoid was highest in school-age children from 5 to 15 years of age, a rate similar to that seen in south-central and southeast Asia [1–3]. There is increasing evidence, that typhoid fever also causes significant morbidity and mortality in children under the age of 5 in these endemic areas [4, 5]. The increased recognition of disease in young children and infants coupled with the emergence of antibiotic resistant strains of *S*. Typhi has renewed interest in development of new vaccines with improved protection over that provided by available licensed vaccines [6, 7].

The widely available typhoid vaccines include a multi-dose, live-attenuated oral vaccine (Vivotif) licensed for people >6 years of age and subunit, parenteral vaccines containing the *S*. Typhi Vi polysaccharide (PS) capsule (Typhim Vi, Typherix, Typbar) licensed for individuals >2 years of age. Both the oral and parenteral vaccines are well-tolerated but require reimmunization every 2–6 years [8–10]. The increased recognition of typhoid fever in children under the age of 5 coupled with the requisite re immunization cycle has catalyzed the development of Vi PS conjugate vaccines with enhanced immunopotency. Anti-Vi IgG levels have been shown to correlate with protection from typhoid fever [11]. Two Vi PS conjugate vaccines comprised of Vi PS conjugated to tetanus toxoid—PedaTyph (Bio Med) and Typbar-TCV (Bharat Biotech) elicit protective levels of anti-Vi antibody titers in adults and children [12, 13]. Typbar-TCV, which contains 25 μg of conjugated Vi PS, is licensed in India and Nepal, and was prequalified by WHO for use in infants >6 months, children, and adults in January 2018, whereas PedaTyph is licensed only in India [14]. Three other Vi conjugate vaccine candidates are at various stages of clinical development. The National Institutes of Health developed a vaccine candidate consisting of Vi PS conjugated to a recombinant *Pseudomonas aeruginosa* exoprotein A (Vi-rEPA). This vaccine was well tolerated and elicited protective levels of anti-Vi antibody titers in adults, children, and infants [15–18] having completed Phase 2 evaluation. Novartis Vaccine Institute for Global Health (NVGH) demonstrated that Vi PS conjugated to CRM197, a genetic toxoid of diphtheria toxin [19, 20], was safe and immunogenic in Phase 1 and 2 trials [21]. The International Vaccine Institute (IVI) has also demonstrated a favorable tolerability and immunogenicity profile of their conjugate vaccine consisting of Vi PS conjugated to diphtheria toxoid in Phase 1 [22]. Although PS conjugate vaccines have proven highly efficacious in the prevention of several bacterial diseases, traditional conjugation technologies like those utilized in the synthesis of the typhoid fever conjugate vaccines can be inefficient resulting in complex manufacturing and increased cost of goods (COG).

Matrivax has developed a simpler, alternative ‘virtual conjugate’ vaccine platform technology termed Protein Capsular Matrix Vaccine (PCMV) [23]. In contrast to conjugation, the PS antigens in a PCMV are non-covalently entrapped in a cross-linked protein matrix. We applied PCMV technology to synthesize a typhoid fever vaccine (Typhax) comprised of Vi PS antigen purified from *S*. Typhi entrapped in a glutaraldehyde catalyzed matrix of cross-linked α-poly-L-lysine (α-PLL) and CRM197 protein. The resulting particles had a radius of ~40 nm, a molecular weight of 6 x 10^6^ Da, and likely consist of 1–2 chains of Vi polysaccharide and 10–20 molecules of CRM197. Each chain of Vi had a molecular weight of 1–2 x 10^6^ Da as determined by size exclusion chromatography and multi-angle laser light scattering. A description of the in vitro characterization, manufacture and preclinical immunogenicity of Typhax was previously reported [24]. Here we describe the results from a Phase 1, ascending dose-response, first-in-human, clinical trial to determine the safety and immunogenicity profile of Typhax.

## Materials and methods

### Ethics statement

Human subject research IRB was Quorum Institutional Review Board in Seattle, WA

### Study vaccine

Typhax was manufactured at Walter Reed Army Institute of Research-Pilot Bioproduction Facility (WRAIR-PBF) in Silver Spring, MD. Each 3 mL vial of Typhax drug product (DP) contained 0.7 mL of a sterile solution of Vi PS from *S*. Typhi entrapped in a glutaraldehyde catalyzed matrix of cross-linked α-PLL and CRM197 protein (Pfenex, Inc.; San Diego, CA). The concentration of Vi PS and CRM197 in the solution was 25.2 μg/mL and 10.4 μg/mL, respectively [24].

Typhax doses were formulated at the clinical site using the following components: 1) a vial of Typhax; 2) a vial containing 1 mL of a sterile diluent (10 mM phosphate buffer and 4 M NaCl, pH 7.4) manufactured at WRAIR-PBF; 3) a vial of sterile 0.9% saline (Hospira, King of Prussia, PA) also used as a diluent; and 4) Adju-Phos (Sergeant Adjuvants, Clifton, NJ), an aluminum phosphate adjuvant. The preparation of Typhax doses (10.0 μg, 2.5 μg, and 0.5 μg) was performed by an unblinded third party at the study site utilizing aseptic techniques according to a Pharmacy Manual supplied to the clinical site. Typhax doses were mixed for 17 ± 5 hours at 2-8ºC prior to administration. Both diluents were required for formulation to maintain the same level of NaCl and osmolality at all doses. The amount of each component in a 0.5 mL dose of the Typhax final drug production formulations are shown in Table 1. The Typhim Vi used in the study was obtained from Sanofi Pasteur SA and each 0.5 mL dose contained 25 μg of Vi PS. The saline used as the placebo was obtained from Hospira (King of Prussia, PA). Syringe barrels were masked with a sleeve prior to intramuscular administration of vaccines and placebo by blinded clinical site staff.

**Table 1:** Amounts of components in Typhax formulations.

Ingredient	Typhax formulation		
	0.5 μg dose	2.5 μg dose	10.0 μg dose
Vi polysaccharide	0.5 μg	2.5 μg	10.0 μg
CRM197	0.2 μg	1.1 μg	4.3 μg
Sodium phosphate monobasic	6.2 μg	29.3 μg	116 μg
Sodium phosphate dibasic	51.8 μg	245 μg	970 μg
Sodium chloride	4.1 mg	4.1 mg	4.1 mg
Adju-Phos	500 μg	500 μg	500 μg

### Study design

The double-blind, ascending dose cohort study was designed to compare the safety, reactogenicity and immunogenicity of the alum-adjuvanted investigational vaccine Typhax at three different dose levels (0.5, 2.5 and 10 μg of Vi antigen) compared to the FDA licensed Typhoid vaccine Typhim Vi and placebo in healthy adult subjects. The study was conducted at Optimal Research, LLC, Rockville, MD, from March 28, 2016 to February 15, 2017.

Healthy adult subjects (n = 45) aged 18 to 55 years were enrolled in the study. Subjects were excluded from the study with: a current or relevant history of physical or psychiatric illness, any medical disorder that required treatment or make the subject unlikely to complete the study, or any condition in the opinion of the Investigator, that presents undue risk from the investigational product or procedures. Patients were also excluded if they had received previous *S*. Typhi vaccination; pre-existing serum anti-Vi IgG antibody levels above baseline at screening (see below); any infection proven or suspected to be caused by *S*. Typhi within 6 months preceding study vaccination; receipt of blood products or immunoglobulins (including monoclonal antibodies) within 12 months before enrollment through conclusion of the study; significant illness, as judged by the investigator, within 2 weeks of the first dose of investigational vaccine; women who were pregnant or breast-feeding and women of childbearing age not willing to use acceptable birth control measures. Women of child bearing potential also underwent a urine pregnancy test.

Enrolled patients for each dose cohort (n = 15) were randomized on the day of immunization into the Typhax, Typhim Vi, and placebo groups at a ratio of 9:3:3. Prior to administration of study vaccines and placebo, vital signs were measured, blood was taken for clinical laboratory testing and determining serum levels of anti-Vi antibody levels, and baseline reactogenicity determined. Subjects were immunized by IM injection in the deltoid muscle and remained in the clinic for at least 60 minutes post-administration for measurement of vital signs and assessment of reactogenicity. On Day 28, a second dose of study vaccine (or placebo) was administered as described for Day 0. A Safety Monitoring Committee consisting of an independent physician, a CRO Medical Monitor, a Sponsor Medical Monitor, and the Investigator reviewed safety study data prior to dose escalation.

This study was conducted under US IND, and ethical review and approval of the study protocol was obtained from the Quorum Institutional Review Board, Seattle, Washington in accordance with the International Conference on Harmonization, Good Clinical Practice guidelines. All participants provided written informed consent prior to study enrollment and the study was registered on clinicaltrials.gov (NCT03926455).

### Safety assessments

Safety assessments were performed at each clinic visit and included measurement of vital signs and adverse events. Vital signs measured included changes in systolic blood pressure, diastolic blood pressure, heart rate, respiration rate and body temperature. Clinical laboratory tests, including a complete blood count with differential and platelet count, serum chemistries and urinalysis, were performed at Days 7, 28, and 180.

### Reactogenicity assessments

Reactogenicity assessments were performed on days of vaccination (Day 0 and 28) and at each follow up site visits by clinical staff. Subjects assessed reactogenicity between visits until 14 days post-vaccination and recorded data in a diary that was subsequently reviewed by clinical staff. Reactogenicity assessments included both local reactogenicity at the injection site (pain, tenderness, erythema, induration) and systemic reactogenicity (fever, malaise/fatigue, myalgia, headache, nausea, vomiting and arthralgias).

### Statistical data analyses

Data were summarized using descriptive statistics, which included the number of subjects (n), mean, standard deviation (SD), median, minimum value, and maximum value, and where applicable, 95% confidence intervals (CI). Percentages were calculated using the number of subjects within each treatment group. Immunogenicity data and statistics included the geometric mean titer (GMT) and the 95% confidence interval of GMT. Further statistical analysis of immunogenicity data is described below. For categorical variables, the counts and proportions of each value were tabulated by dosage group. Data from subjects randomized to receive Placebo and Typhim Vi were pooled from the 3 cohorts for the purposes of analysis and all subjects received the assigned treatments.

Adverse events (AEs) were tabulated using the Medical Dictionary for Drug Regulatory Affairs (MedDRA) body system and preferred terms [25]. AEs were counted from the first vaccination up to 28 days after the last dose. Subjects were asked if any changes in health status occurred since the last clinic visit. For MedDRA body system, the number and percentage of subjects experiencing at least one AE overall and at least one AE for a body system were tabulated by severity and dosage group. For the MedDRA preferred term, the number and percentages of subjects reporting at least one AE within a preferred term category were tabulated by relationship to study products and dosage group. Each subject’s AEs were counted once under the maximum severity or the strongest recorded causal relationship to study product. No formal statistical testing of the incidence of AEs across treatment groups was performed.

Clinical laboratory data summary statistics (descriptive) were presented by dosage group for all baseline and post-vaccination laboratory values and for changes from baseline measured during the study. The number (percentage) of subjects with post-vaccination local laboratory values recorded as grade 1 AE criteria or above as specified in the Food and Drug Administration (FDA) Center for Biologics Evaluation and Research (CBER) AE Grading Table [18].

For reactogenicity data, the number and percentage of subjects experiencing each type of reactogenicity finding after each vaccination were tabulated by dosage group and severity as graded according to the FDA CBER 2007 Guidance for Industry: Toxicity Grading Scale for Healthy Adult and Adolescent Volunteers Enrolled in Preventive Vaccine Clinical Trials.

### Anti-Vi serum IgG antibody immunogenicity assessments by ELISA

Subjects were screened for pre-existing IgG antibodies to Vi polysaccharide prior to inclusion in the study using an ELISA kit from Alpha Diagnostic International (San Antonio, TX). Subjects were excluded from the study if they exhibited a titer above the cut-off control sera level of 3.6 U/mL.

Serum samples were collected on Days 0, 14, 28, 42 and 180 to determine anti-Vi IgG antibody titers using an endpoint titration enzyme-linked immunosorbent assay (ELISA) by Advanced Bioscience Lab Inc. Rockville, MD. Briefly, Immulon 96 well ELISA plates were coated with a 2 μg/mL solution of Vi PS (Matrivax) in Phosphate Buffered Saline (PBS) and incubated at 2–8°C for a minimum of 16 hours. Following coating, the plates were washed 3 times with PBS containing 0.05% Tween (PBST) to remove unbound coating solution. After washing, plates were blocked using PBST containing 3% BSA at 2-8ºC for 16–72 hours, decanted, and washed 3 times with PBST. Diluted serum samples (1:50 to 1:6400) and positive/negative controls were added and allowed to incubate at 4°C for 16-18 hours. The exception to this was the Day 0, 14, 28, and 42 serum samples for cohort 1 where the dilutions were 1:100 to 1:12,800. After overnight incubation, plates were washed 4 times with PBST and then 100 μl of biotinylated rabbit anti-human IgG (Sigma) diluted 1:10,000 in PBST was added to each well. The plate was placed on a plate shaker set at 200 RPM for 90 to 120 minutes at ambient temperature. After incubation, plates were washed 4 times with PBST and then 100 μl of streptavidin-AP (BD Biosciences) diluted 1:1,000 in PBST was added to each well and placed on a plate shaker set at 200 RPM for 60 to 90 minutes at ambient temperature. After incubation, plates were washed 4 times with PBST, para-Nitrophenylphosphate (pNPP) solution (Sigma) added (100 μL/well) and incubated for 30 minutes at ambient temperature in the dark. Plates were read at a wavelength of 405 nm on a plate reader.

The anti-Vi IgG antibody endpoint titer was determined by using endpoint titer cutoff method [26]. The endpoint titer cutoff was defined as the A_405_ value that is two standard deviations above the mean of the negative control serum sample. For samples where the A_405_ value of the starting dilution was below the cutoff, the titer was reported as half that dilution. For example, if the initial dilution was 50, the titer would be reported as 25. The individual endpoint titers for each human serum in an experimental group were then used to calculate the GMT for each experimental group in the study. Negative human serum and a positive reference control Vi antiserum was also run on each plate to validate the assay performance. The human sera used as positive and negative controls were obtained from BioreclamationIVT and the anti-Vi titer determined at Matrivax prior to being provided to Advanced Bioscience Lab Inc for use in analyzing clinical samples. A positive immune response (seroconversion) by ELISA was defined as at least a 4-fold increase over baseline in the Vi-specific IgG antibody titer as measured by ELISA. Titers between vaccine and placebo groups were compared using a Mann Whitney test. Titers within a group from Day 14 to Day 180 post initial vaccination were compared using a paired ANOVA (Friedman’s test] with a Dunn’s multiple comparisons post test.

## Results

### Subject demographics and treatment assignment

**Fig 1:**
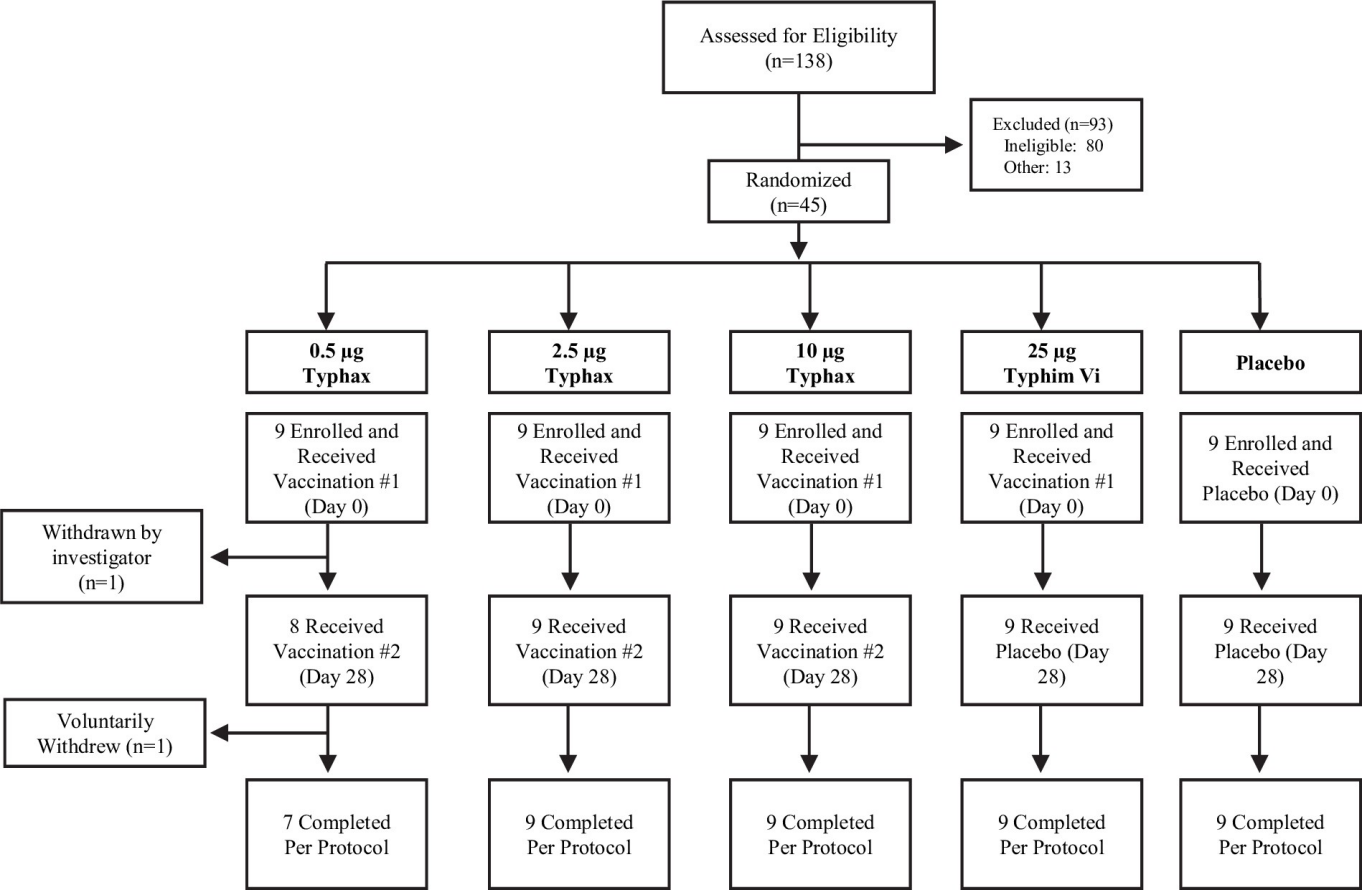
Subject completion flow chart.

Of the 138 subjects screened for the Phase 1 trial, 80 were ineligible based on the inclusion and exclusion criteria and 13 others because enrollment had closed (Fig 1). The majority of the 80 subjects deemed ineligible were due to an inability to comply with study procedures, generally considered unhealthy, or had laboratory parameters that were considered clinically significant. Seventeen [17] of these 80 subjects were excluded based on anti-Vi IgG levels greater than the cut-off value (3.6 U/mL) from the ELISA screen. The 45 subjects enrolled in trial consisted of 23 (51.1%) male and 22 (48.9%) female subjects with a mean ± SD age of 38.9 ± 11.3 years. Complete demographic characteristics of the study population is provided in supplemental information (S1 Table). Minor differences in demographics across treatment groups did not appear to impact the overall results of the study.

Enrolled patients (n = 15/cohort) were randomized at the time of the first immunization into the Typhax, Typhim Vi, and placebo groups at a ratio of 9:3:3. For the 0.5 μg Typhax dose group, two patients failed to complete the study per protocol; 1) relocated and missed Day 180 timepoint and 2) withdrawn by investigator due to inappropriate behavior toward clinical staff, thus no immunogenicity samples were collected (Fig 1). For all other cohorts, all patients completed the study per protocol.

At the time of Typhax manufacture it was unclear from preclinical studies whether an adjuvant was required for optimal immunogenicity, so the drug product was filled unadjuvanted into vials. Prior to start of the toxicology study and clinical trial, however, we demonstrated that optimal immunogenicity with Typhax was obtained when adjuvanted with aluminum phosphate (Adju-Phos) for a minimum of 12 hours for adsorption to the adjuvant to reach equilibrium. Therefore, for the clinical trial, Typhax was adjuvanted by unblinded clinical staff 17 ± 5 hours prior to immunization.

### Safety results: Adverse events (AEs)

The incidence of AEs reported in the study are summarized in Table 2. All AEs were mild to moderate intensity, except for a single case of severe anemia reported in the Placebo group. There were no remarkable differences in AEs between the Typhax dose groups and the Typhim Vi and Placebo groups. The treatment-emergent AE (TEAEs) considered to be related (possibly, probably, definitely) to study drug were dizziness (Typhim Vi, n = 1; immediately after first immunization), myalgia (Typhim Vi, n = 1; 7 days after first immunization), injection site bruising (Typhax 10.0 μg, n = 1; 1 day after second immunization), and nasopharyngitis (Typhax 10.0 μg, n = 1; 2 days after second immunization). There were two reported serious adverse events (SAEs), neither of which was considered by the Investigator or Sponsor to be related to administration of study vaccine. The reported SAEs were psychiatric disorder (one subject in the 2.5 μg Typhax group) group and an appendectomy (one subject in the 10.0 μg Typhax group) that occurred on Day 7 of study. Both patients recovered and completed the study. Collectively, there were no remarkable changes from baseline in mean values in blood pressure, pulse rate, and body temperature post vaccination. Additionally, there were no observed changes in laboratory tests during the study that were considered attributable to administration of study medication.

**Table 2:** Summary of adverse events.

Number of Subjects with TEAE	Typhax 0.5 μg (N = 9)	Typhax 2.5 μg (N = 9)	Typhax 10.0 μg (N = 9)	Typhim Vi 25.0 μg (N = 9)	Placebo (N = 9)
**Any TEAE**	2 (22.2)	3 (33.3)	4 (44.4)	5 (55.6)	5 (55.6)
**Serious TEAE**	0	1 (11.1)^b^	1 (11.1)^c^	0	0
**Study Drug-Related TEAE**^a^	0	0	2 (22.2)^d^	2 (22.2)^e^	0
**Study Drug-Related Serious TEAE**^a^	0	0	0	0	0
**Grade 3 or 4 TEAE**	0	0	0	0	1 (11.1)
**TEAE Leading to Dose Discontinuation**	0	0	0	0	0
**Non-TEAE**	0	0	1 (11.1)	0	0

TEAE, treatment-emergent adverse event

^a^Drug-Related TEAEs include Possibly Related, Probably Related, and Definitely Related. Subjects are counted once for each category. A subject may be in more than one overall summary.

^b^Psychiatric disorder

^c^Appendectomy

^d^Injection site bruising (n = 1), nasopharyngitis (n = 1)

^e^Dizziness (n = 1), myalgia (n = 1)

### Reactogenicity results

Local and Systemic Reactogenicity after the first and second injections are summarized in Table 3. After the first dose, more than half the subjects in Typhax and Typhim Vi groups reported local symptoms at the injection site mainly attributed to pain and tenderness whereas most subjects treated with Placebo did not report these findings. There was a dose related increase in the incidence and severity in pain and tenderness at the injection site with Typhax treatment. Most reports of pain and tenderness at the injection site were classified as grade 0, 1, or grade 2 in severity, with one subject in the Typhax 10 μg group having both pain and tenderness rated as grade 3. Except for one subject in the Placebo group who reported a mild erythema, none of subjects in the Typhax or Typhim Vi vaccine groups reported any symptom of erythema or induration.

**Table 3:** Summary of local injection site and systemic symptoms within 14 days after first and second vaccination by grade.

Symptom Type	Grade	Typhax 0.5 μg (N = 9)	Typhax 2.5 μg (N = 9)	Typhax 10.0 μg (N = 9)	Typhim Vi 25.0 μg (N = 9)	Placebo (N = 9)
**Local Symptoms after First Injection and before Second Injection**^a^						
**Any**	0	3 (33.3)	3 (33.3)	0	2 (22.2)	7 (77.8)
	1	6 (66.7)	5 (55.6)	6 (66.7)	7 (77.8)	2 (22.2)
	2	0	1 (11.1)	2 (22.2)	0	0
	3	0	0	1 (11.1)	0	0
	4	0	0	0	0	0
**Systemic Symptoms after First Injection and before Second Injection**^b^						
**Any**	0	4 (44.4)	5 (55.6)	4 (44.4)	6 (66.7)	6 (66.7)
	1	3 (33.3)	2 (22.2)	4 (44.4)	3 (33.3)	3 (33.3)
	2	2 (22.2)	2 (22.2)	1 (11.1)	0	0
	3	0	0	0	0	0
	4	0	0	0	0	0
**Local Symptoms after Second Injection**^a^						
**Any**	0	4 (44.4)	1 (11.1)	3 (33.3)	7 (77.8)	8 (88.9)
	1	4 (44.4)	8 (88.9)	4 (44.4)	2 (22.2)	1 (11.1)
	2	0	0	2 (22.2)	0	0
	3	0	0	0	0	0
	4	0	0	0	0	0
**Systemic Symptoms after Second Injection**^b^						
**Any**	0	4 (44.4)	9 (100)	3 (33.3)	8 (88.9)	7 (77.8)
	1	3 (33.3)	0	5 (55.6)	1 (11.1)	1 (11.1)
	2	1 (11.1)	0	1 (11.1)	0	1 (11.1)
	3	0	0	0	0	0
	4	0	0	0	0	0

^a^Local Symptoms include: pain, tenderness, erythema (redness), and induration (swelling).

^b^Systemic Symptoms include: fever, headache, arthralgia (joint pain), joint swelling, malaise/fatigue, myalgia (muscle ache), nausea, vomiting, and diarrhea.

Systemic symptoms following the first injection were predominantly grade 1 headache, fatigue and myalgia. Fever was reported in the Placebo group (grade 1) and in the 10.0 μg Typhax group (grade 2). One subject in the 0.5 μg Typhax group reported nausea, diarrhea and fatigue, all grade 2. Except for one episode of grade 1 joint pain in the 2.5 μg Typhax group, there were no symptoms of joint swelling or vomiting in any treatment group. One to 3 subjects in each Typhax group reported nausea and diarrhea, however the incidence and severity of any symptom among the Typhax groups was not dose related.

There were fewer reports of both local and systemic symptoms following the second injection. The incidences of local pain and tenderness at the injection site improved with all subjects reporting no pain symptoms or grade 1 intensity. The maximal intensity of pain and tenderness in the 10.0 μg Typhax group decreased to grade 1 and 2, respectively. There were no grade 3 local symptoms reported and no incidences of erythema or induration. Systemically, a general improvement in symptoms was observed as more subjects reported no symptoms [31 of 44; one subject did not receive the second injection), 10 reported grade 1 symptoms and three subjects reported grade 2 symptoms). There were slight decreases in the incidence of myalgia, nausea, and fatigue. Fever of grade 1 intensity was also noted after the second dose of 10.0 μg Typhax, but also after the Placebo injection (grade 1, n = 1 and grade 2, n = 1). This finding suggests that fever is not an uncommon event, especially in the outpatient setting. There were no differences in the severity-by-duration of systemic symptom scores between the Typhax vaccinations and Typhim Vi or Placebo. The incidence and severity of tenderness was slightly higher with 10 μg Typhax compared to the other treatment groups. The results also demonstrated that the second dose of Typhax (Day 28) was not associated with increased reactogenicity.

### Immunogenicity results: Anti-Vi IgG antibody ELISA titers

**Fig 2:**
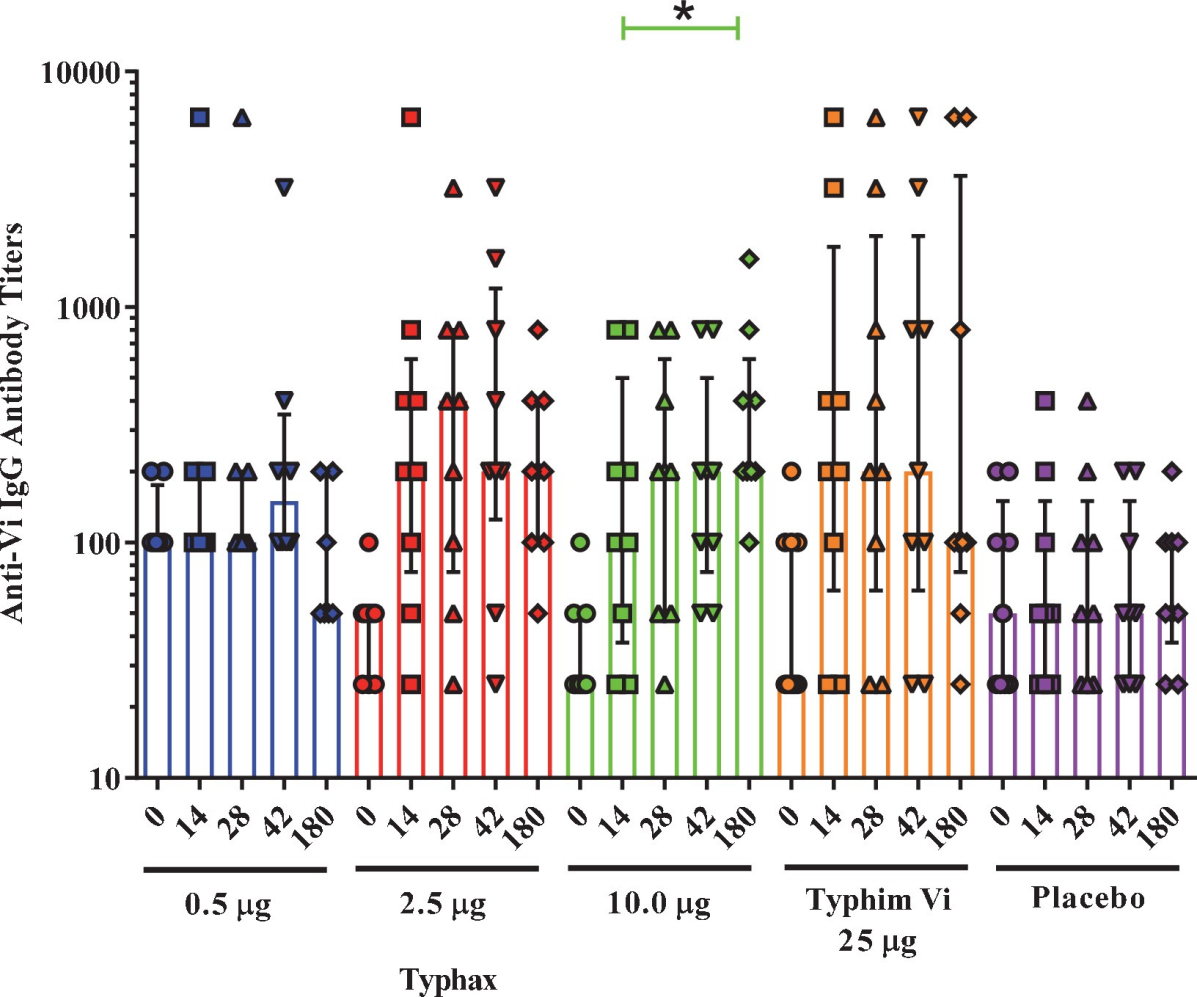
Anti-Vi IgG titers of subjects immunized with Typhax, Typhim Vi or Placebo. For Typhax groups, subjects were immunized on Day 0 and Day 28, whereas in the Typhim Vi group, subjects were immunized with Typhim Vi on Day 0 and a placebo on Day 28. Anti-Vi IgG titers were determined for each subject at Day 0, 14, 28, 42, and 180 and the individual titers represented by a symbol. The median titers of each group at each timepoint is shown by the box and the bars represent the 25^th^ and 75^th^ quartiles. Antibody titers for each vaccine group following the first immunization (Day 14 to Day 180) were compared over time using a paired ANOVA (Friedman’s test) with a Dunn’s multiple comparison post test. The line with an asterisk (*) indicates a significant difference in titers in the 10 μg dose group between the Day 14 and Day 180 in the post test (p value = 0.015).

Blood samples were drawn from each patient on Day 0 (pre-vaccination), 14, 28, 42, and 180 (6 months). The anti-Vi IgG serum level for each patient across all time points was determined by end point titration ELISA (Fig 2 and S2–S4 Tables). The anti-Vi IgG GMT for Day 0, 14, 28, and 42 serum samples for each cohort were determined after the Day 42 samples were collected (Table 4). The Day 180 serum samples for all three cohorts were analyzed at the same time along with a remeasurement of the Day 0 serum samples. Similar GMTs were observed with both measurements of the Day 0 samples for all Typhax groups except for the 0.5 μg Typhax group where the GMT decreased from 119 to 45 (S2–S4 Tables). This difference was likely due to a change in the starting dilution of sera used in the analysis of the Day 0, 14, 28, and 42 serum samples of cohort 1 (1:100) and the starting dilution used in the analysis of all subsequent cohorts and Day 180 measurements (1:50). Since the baseline titer for non-responders is reported as half the starting dilution, using a 1:100 starting dilution for the 0.5 μg Typhax cohort resulted in a baseline titer of 50 rather than 25 and may have artificially inflated the GMTs at the Day 0, 14, 28, and 42 time points. Similarly, the GMT of the Day 0 sample was different for the Typhim Vi and Placebo groups between the analyses (S2 Table).

**Table 4:** Anti-Vi IgG serum antibody geometric mean titers and percent seroconversion.

Time Point		Typhax			Typhim Vi	Placebo
		0.5 μg	2.5 μg	10.0 μg	25 μg	
Day 0	N	8	9	9	9	9
	GMT (95% CI)	119 (90, 156)	43 (30, 61)	34 (23, 50)	50 (26, 96)	58 (29, 117)
	Seroconversion, n (%)	0 (0)	0 (0)	0 (0)	0 (0)	0 (0)
Day 14	N	8	9	9	9	9
	GMT (95% CI)	200 (60, 664)*	252 (72, 879)	126 (47, 341)	272 (63, 1174)	63 (30, 134)
	Seroconversion, n (%)	1 (12.5)	7 (77.8)	5 (55.6)	5 (55.6)	0 (0)
Day 28	N	8	9	9	9	9
	GMT (95% CI)	200 (60, 664)	272 (85, 872)*	171 (66, 444)	294 (67, 1299)	68 (32, 145)
	Seroconversion, n (%)	1 (12.5)	7 (77.8)	6 (66.7)	5 (55.6)	0 (0)
Day 42	N	8	9	9	9	9
	GMT (95% CI)	218 (80, 593)*	294 (89, 971)*	171 (78, 378)*	294 (63, 1361)	58 (31, 111)
	Seroconversion, n (%)	2 (25)	7 (77.8)	6 (66.7)	5 (55.6)	0 (0)
Day 180	N	7	9	9	9	9
	GMT (95% CI)	82 (45, 151)	200 (104, 384)*	318 (165, 610)*	252 (52, 1219)	63 (35, 114)
	Seroconversion, n (%)	2 (28.6)	5 (55.6)	7 (77.8)	5 (55.6)	0 (0)

*p value <0.05 by Mann-Whitney U test compared to Placebo

Two weeks following the first immunization (Day 14), anti-Vi GMTs increased from the baseline antibody levels in the 2.5 and 10 μg Typhax and Typhim Vi groups. The titers, however, did not increase further at Day 28 for any group (Table 4 and Fig 2). Administration of a second Typhax dose on Day 28 did not result in a measurable increase in GMT at Day 42 or Day 180 at any dose level, except for the 10.0 μg Typhax group, where the titer increased from 171 to 318, however this increase did not reach statistical significance (Table 4 and Fig 2). However, when the titers for each vaccine groups were compared across all time points following the first immunization (Day 14 to Day 180) using a paired ANOVA (Friedman’s test), the only statistically significant change was found in the 10 μg dose group between the Day 14 and Day 180 timepoints (p value = 0.015) (Fig 2).

After a single administration, all dose levels of Typhax and Typhim Vi elicited similar levels of anti-Vi titers (Table 4). The seroconversion rate for the 2.5 μg and 10.0 μg Typhax dose groups from Day 14 through Day 180 were 55.6% to 77.8% while the Typhim Vi group remained at 55.6% throughout all time points. The 0.5 μg Typhax dose group, while eliciting similar anti-Vi IgG antibody titers, was relatively ineffective at seroconversion (1 or 2 subjects) whereas subjects in the placebo group did not seroconvert.

## Discussion

Overall, this active- and placebo-controlled, Phase 1 trial demonstrated that Typhax was safe, well tolerated, and immunogenic. The incidence and severity of the reported AEs were similar in Typhax and Typhim Vi immunized subjects and there were no observed changes in clinical laboratory tests during the study that were considered attributable to administration of Typhax. However, there was a dose related increase in the incidence and severity in pain and tenderness at the injection site following the first immunization with Typhax, although the incidence and severity decreased following the second immunization.

The immunogenicity data indicate that Typhax elicited an immune response that is comparable to the licensed vaccine Typhim Vi although at lower Vi PS amounts. Following a single administration of vaccines, the 2.5 and 10 μg doses of Typhax and Typhim Vi elicited an increase in anti-Vi IgG GMT above baseline (pre-immunization) at Day 14. Antibody titers from the Typhax dose groups and Typhim Vi, however, did not increase significantly at Day 28. Despite similar GMTs elicited by all Typhax dose levels, the seroconversion rates observed at the 2.5 μg and 10.0 μg dose levels (77.8% and 55.6%, respectively) were substantially higher than that observed at the 0.5 μg dose level (12.5%) and similar to what was observed with 25 μg of Typhim Vi (55.6%). The GMT reported for the 0.5 μg dose at the Day 0, 14, 28, and 42, however, may have been artificially high due to the procedure used to determine the baseline titer for non-responders for this cohort.

In preclinical animal studies, both Typhax and Vi conjugates elicited increases in anti-Vi IgG titers following a second dose [16, 24]. However, in clinical studies in healthy adults, neither Typhax nor Vi conjugates elicited a significant increase in anti-Vi IgG antibody titers 1- to 4-weeks following a second administration [16, 22]. However, increases in anti-Vi responses were observed in 2–4 year old children with the Vi-rEPA conjugate vaccine following a second dose 6 weeks after the initial immunization [15] and with Typbar-TCV 720 days following the initial dose [12]. Together these data suggest that Vi conjugates can elicit a memory response although the timing of the second dose and age of the patient may be factors. While the data from this study do not indicate that Typhax elicited an immediate anamnestic immune response, a slight but statistically significant increase in titer was observed with the 10 μg Typhax dose regimen at Day 180 compared to the titer seen at Day 14 (two weeks following first dose). This potential delay in immune response at the 10 μg dose could be due to the particulate nature of the Typhax particles or the fact that it is adjuvanted with Adju-Phos which may result in a slower release of antigen due to adsorption to the adjuvant. Unlike Vi conjugates [27], preclinical studies in mice and rabbits demonstrated that Typhax required an adjuvant to elicit a more robust immune response [24].

In humans, Typhax at all dose levels elicited similar antibody titers to the unconjugated Vi PS. This contrasts with what has been reported for Vi conjugates which elicited superior titers compared to unconjugated Vi PS [12, 15, 16, 21, 22, 28]. However, like the Vi conjugates, Typhax at the 2.5 and 10 μg dose levels elicited similar rates of seroconversion as unconjugated Vi polysaccharide [12, 21, 22]. The rate of seroconversion, however, for the unconjugated Vi polysaccharide comparator in our study was 55% compared to 80–90% in the Vi conjugate studies [12, 16, 21, 22]. The reported seroconversion rates for Typhim Vi varied with clinical studies, age of subjects, and time post immunization, but was between 62.5% and 100% [29, 30]. The lower than expected seroconversion rate observed in our study could be due to the ELISA procedure used for determining anti-Vi IgG titers as well as the low number of subjects used in this study.

The results from this trial support continued clinical evaluation of Typhax as a vaccine candidate for the prevention of typhoid fever. Typhoid fever remains a significant health problem in Africa, the Indian subcontinent, and Southeast Asia. Although school age children (5 to 15 years of age) represent the largest age group impacted by typhoid fever, there is increased recognition that typhoid fever is also a significant cause of disease in children less than 5 years of age and infants (4]. Unfortunately, the widely available commercial vaccines (Typhim Vi, Typherix, and Vivotif) are not approved for young children and infants thus prompting the development of Vi conjugate vaccines to protect this vulnerable age group. There are now two Vi-tetanus toxoid conjugates (Typbar-TCV and PedaTyph) approved for use in children and adults and Typbar-TCV has recently gained WHO prequalification for use in infants >6 months of age in India and Nepal.

As an alternative to traditional conjugation technology, Matrivax is developing PCMV technology for the synthesis of polysaccharide-based vaccines. Conjugation requires polysaccharide antigen resizing, derivatization and covalent cross-linking to a protein carrier molecule. In contrast, PCMV technology entraps polysaccharide antigens in a cross-linked matrix of protein in a process that does not modify the structure of the protective full-length polysaccharide antigen. In preclinical animal studies, PCMVs have proven to elicit high titer polysaccharide specific IgG antibodies and an anamnestic immune response similar to that observed with conjugate vaccines [23, 24]. It has been suggested, however, that non-conjugated polysaccharide-based vaccines, like PCMVs, that elicit high titers in preclinical animal studies are non-immunogenic in humans [31, 32]. The results from this Phase 1 clinical trial clearly demonstrate that Typhax, an alum-adjuvanted Vi PS PCMV, can elicit similar antibody titers and seroconversion rates as the licensed unconjugated Vi polysaccharide vaccine at lower amounts of Vi polysaccharide. Overall, the results from this study, show that Typhax is safe, well tolerated, and immunogenic in healthy adults and supports the further clinical development of Typhax.

There are several limitations to the current study that should be considered in interpreting the data. While the antibody titer data indicate that Typhax and Typhim Vi elicited increased titers relative to placebo the number of subjects immunized in each group was insufficient to adequately compare groups. Furthermore, based on the amount and concentration of Typhax, we were not able to administer an equivalent amount of Vi polysaccharide to patients as Typhim Vi (25 μg) for a direct comparison nor did we compare the immune response to a Vi conjugate vaccine. Lastly, the higher initial dilution of sera from 0.5 μg Typhax group used in the ELISA to determine anti-Vi titers during resulted in higher baseline titers of non-responders likely resulted in an overestimate of antibody titers in this group at Day 0, 14, 28, and 42. In future clinical assessments of Typhax efforts will be made to standardize the ELISA procedure and to obtain common reference sera in order to compare antibody responses to those seen with other Vi polysaccharide containing vaccines.

## Supporting information

S1 Checklist CONSORT checklist. (DOC)

S1 Table Demographic characteristics of study subjects. (DOCX)

S2 Table Immunogenicity data from Cohort 1. (DOCX)

S3 Table Immunogenicity data from Cohort 2. (DOCX)

S4 Table Immunogenicity data from Cohort 3. (DOCX)
