# Supplementary material for: A phase 1 randomized safety, reactogenicity, and immunogenicity study of Typhax: A novel protein capsular matrix vaccine candidate for the prevention of typhoid fever
Source: PLoS Negl Trop Dis. 2020 Jan 6;14(1):e0007912. doi: 10.1371/journal.pntd.0007912 (PMC6964911; doi:10.1371/journal.pntd.0007912)
Supplement: S1 Table — (DOCX) [file pntd.0007912.s002.docx]

| **Table S1. Demographic Characteristics of Study Subjects** | | | | | | |
| --- | --- | --- | --- | --- | --- | --- |
| **Parameter** | **Typhax** | | | **Typhim Vi** | **Placebo** | **Total** |
| **Category or Statistic** | **0.5 µg** | **2.5 µg** | **10.0 µg** | **25.0 µg** |  |  |
| **Age, years** | | | | | | |
| N | 9 | 9 | 9 | 9 | 9 | 45 |
| Mean (SD) | 39.6 (13.71) | 37.6 (12.63) | 33.3 (7.73) | 45.0 (6.12) | 38.9 (13.27) | 38.9 (11.28) |
| Median | 43 | 43 | 36 | 46 | 35 | 43 |
| Min, Max | 22, 54 | 20, 53 | 24, 47 | 30, 51 | 22, 55 | 20, 55 |
| **Gender, n (%)** |  |  |  |  |  |  |
| Female | 4 (44.4) | 5 (55.6) | 5 (55.6) | 1 (11.1) | 7 (77.8) | 22 (48.9) |
| Male | 5 (55.6) | 4 (44.4) | 4 (44.4) | 8 (88.9) | 2 (22.2) | 23 (51.1) |
| **Ethnicity** | | | | | | |
| Non-Hispanic or Latino | 9 (100) | 8 (88.9) | 7 (77.8) | 9 (100) | 8 (88.9) | 41 (91.1) |
| Hispanic or Latino | 0 | 1 (11.1) | 2 (22.2) | 0 | 1 (11.1) | 4 (8.9) |
| **Race, n (%)** | | | | | | |
| White | 3 (33.3) | 2 (22.2) | 6 (66.7) | 2 (22.2) | 4 (44.4) | 17 (37.8) |
| Black or African-American | 6 (66.7) | 6 (66.7) | 2 (22.2) | 6 (66.7) | 5 (55.6) | 25 (55.6) |
| Asian | 0 | 1 (11.1) | 0 | 0 | 0 | 1 (2.2) |
| American Indian or Alaskan Native | 0 | 0 | 0 | 1 (11.1) | 0 | 1 (2.2) |
| Multiple | 0 | 0 | 1 (11.1) | 0 | 0 | 1 (2.2) |
| **Height, cm** | | | | | | |
| N | 9 | 9 | 9 | 9 | 9 | 45 |
| Mean (SD) | 172.3 (7.68) | 168.2 (4.60) | 168.3 (10.6) | 173.08 (7.22) | 165.9 (8.00) | 169.6 (7.98) |
| Median | 172.72 | 170 | 166 | 170 | 165 | 170 |
| Min, Max | 163.0, 188.0 | 160.0, 173.0 | 150.0, 182.9 | 163.0, 183.0 | 155.0, 183.0 | 150.0, 188.0 |
| **Weight, kg** | | | | | | |
| N | 9 | 9 | 9 | 9 | 9 | 45 |
| Mean (SD) | 78.13 (17.2) | 83.00 (7.63) | 82.16 (13.6) | 84.30 (15.1) | 87.78 (19.6) | 83.07 (14.8) |
| Median | 75 | 83 | 82 | 77 | 90 | 81 |
| Min, Max | 61.0, 107.0 | 71.0, 96.0 | 64.0, 98.0 | 74.0, 111.0 | 56.0, 121.0 | 56.0, 121.0 |
