# Supplementary material for: A phase 1 randomized safety, reactogenicity, and immunogenicity study of Typhax: A novel protein capsular matrix vaccine candidate for the prevention of typhoid fever
Source: PLoS Negl Trop Dis. 2020 Jan 6;14(1):e0007912. doi: 10.1371/journal.pntd.0007912 (PMC6964911; doi:10.1371/journal.pntd.0007912)
Supplement: S2 Table — (DOCX) [file pntd.0007912.s003.docx]

| **Table S2. Immunogenicity data from Cohort 1** | | | | | | | | |
| --- | --- | --- | --- | --- | --- | --- | --- | --- |
| **Cohort** | **Group** | **Subject ID** | **Anti-Vi IgG titer-1** | | | | **Anti-Vi IgG Titer-2** | |
|  |  |  | **Day 0** | **Day 14** | **Day 28** | **Day 42** | **Day 0** | **Day 180** |
| 1 | Typhax  0.5 µg | 100-007 | 100 | 100 | 100 | 100 | 25 | 50 |
|  |  | 100-008 | Subject Withdrawn by Investigator | | | | | |
|  |  | 100-009 | 100 | 100 | 100 | 100 | 50 | 50 |
|  |  | 100-011 | 100 | 100 | 100 | 200 | 25 | 100 |
|  |  | 100-024 | 200 | 6400 | 6400 | 3200 | Subject Relocated | |
|  |  | 100-034 | 100 | 200 | 200 | 100 | 50 | 200 |
|  |  | 100-040 | 100 | 100 | 100 | 100 | 25 | 50 |
|  |  | 100-044 | 100 | 100 | 100 | 400 | 50 | 50 |
|  |  | 100-046 | 200 | 200 | 200 | 200 | 200 | 200 |
|  |  | **GMT** | **119** | **200** | **200** | **218** | **45** | **82** |
|  |  | **Mean** | **125** | **912** | **912** | **550** | **61** | **100** |
|  |  | **Median** | **100** | **100** | **100** | **150** | **50** | **50** |
|  | Typhim Vi  25 µg | 100-003 | 200 | 6400 | 6400 | 6400 | 200 | 6400 |
|  |  | 100-004 | 100 | 100 | 100 | 100 | 25 | 100 |
|  |  | 100-032 | 100 | 200 | 200 | 200 | 50 | 100 |
|  |  | **GMT** | **126** | **504** | **504** | **504** | **63** | **400** |
|  |  | **Mean** | **133** | **2233** | **2233** | **2233** | **92** | **2200** |
|  |  | **Median** | **100** | **200** | **200** | **200** | **50** | **100** |
|  | Placebo | 100-006 | 100 | 100 | 100 | 100 | 25 | 50 |
|  |  | 100-012 | 200 | 200 | 400 | 200 | 200 | 200 |
|  |  | 100-049 | 200 | 400 | 200 | 200 | 100 | 100 |
|  |  | **GMT** | **159** | **200** | **200** | **159** | **79** | **100** |
|  |  | **Mean** | **167** | **233** | **233** | **167** | **108** | **117** |
|  |  | **Median** | **200** | **200** | **200** | **200** | **100** | **100** |
