# Supplementary material for: A phase 1 randomized safety, reactogenicity, and immunogenicity study of Typhax: A novel protein capsular matrix vaccine candidate for the prevention of typhoid fever
Source: PLoS Negl Trop Dis. 2020 Jan 6;14(1):e0007912. doi: 10.1371/journal.pntd.0007912 (PMC6964911; doi:10.1371/journal.pntd.0007912)
Supplement: S3 Table — (DOCX) [file pntd.0007912.s004.docx]

| **Table S3. Immunogenicity data from Cohort 2** | | | | | | | | |
| --- | --- | --- | --- | --- | --- | --- | --- | --- |
| **Cohort** | **Group** | **Subject ID** | **Anti-Vi IgG titer-1** | | | | **Anti-Vi IgG Titer-2** | |
|  |  |  | **Day 0** | **Day 14** | **Day 28** | **Day 42** | **Day 0** | **Day 180** |
| 2 | Typhax  2.5 µg | 100-058 | 25 | 25 | 25 | 25 | 25 | 50 |
|  |  | 100-061 | 50 | 800 | 800 | 1600 | 50 | 200 |
|  |  | 100-067 | 25 | 100 | 100 | 200 | 100 | 200 |
|  |  | 100-070 | 50 | 200 | 400 | 200 | 100 | 200 |
|  |  | 100-074 | 100 | 400 | 400 | 400 | 100 | 400 |
|  |  | 100-085 | 25 | 50 | 50 | 50 | 25 | 100 |
|  |  | 100-086 | 50 | 200 | 200 | 200 | 50 | 100 |
|  |  | 100-088 | 50 | 400 | 800 | 800 | 50 | 400 |
|  |  | 100-090 | 50 | 6400 | 3200 | 3200 | 50 | 800 |
|  |  | **GMT** | **43** | **252** | **272** | **294** | **54** | **200** |
|  |  | **Mean** | **47** | **953** | **664** | **742** | **61** | **272** |
|  |  | **Median** | **50** | **200** | **400** | **200** | **50** | **200** |
|  | Typhim Vi  25 µg | 100-052 | 100 | 400 | 400 | 800 | 50 | 100 |
|  |  | 100-084 | 25 | 25 | 25 | 25 | 25 | 25 |
|  |  | 100-087 | 25 | 400 | 800 | 800 | 50 | 800 |
|  |  | **GMT** | **40** | **159** | **200** | **252** | **40** | **126** |
|  |  | **Mean** | **50** | **275** | **408** | **542** | **42** | **308** |
|  |  | **Median** | **25** | **400** | **400** | **800** | **50** | **100** |
|  | Placebo | 100-019 | 25 | 25 | 25 | 25 | 100 | 100 |
|  |  | 100-063 | 100 | 50 | 100 | 50 | 100 | 50 |
|  |  | 100-072 | 50 | 50 | 50 | 50 | 100 | 100 |
|  |  | **GMT** | **50** | **40** | **50** | **40** | **100** | **79** |
|  |  | **Mean** | **58** | **42** | **58** | **42** | **100** | **83** |
|  |  | **Median** | **50** | **50** | **50** | **50** | **100** | **100** |
