# Supplementary material for: A phase 1 randomized safety, reactogenicity, and immunogenicity study of Typhax: A novel protein capsular matrix vaccine candidate for the prevention of typhoid fever
Source: PLoS Negl Trop Dis. 2020 Jan 6;14(1):e0007912. doi: 10.1371/journal.pntd.0007912 (PMC6964911; doi:10.1371/journal.pntd.0007912)
Supplement: S4 Table — (DOCX) [file pntd.0007912.s005.docx]

| **Table S4. Immunogenicity data from Cohort 3** | | | | | | | | |
| --- | --- | --- | --- | --- | --- | --- | --- | --- |
| **Cohort** | **Group** | **Subject ID** | **Anti-Vi IgG titer-1** | | | | **Anti-Vi IgG Titer-2** | |
|  |  |  | **Day 0** | **Day 14** | **Day 28** | **Day 42** | **Day 0** | **Day 180** |
| 3 | Typhax  10 µg | 100-106 | 25 | 25 | 50 | 100 | 25 | 200 |
|  |  | 100-109 | 25 | 25 | 25 | 50 | 50 | 200 |
|  |  | 100-113 | 25 | 800 | 800 | 800 | 25 | 1600 |
|  |  | 100-115 | 25 | 200 | 200 | 200 | 50 | 200 |
|  |  | 100-120 | 50 | 800 | 800 | 800 | 100 | 800 |
|  |  | 100-124 | 25 | 100 | 200 | 200 | 25 | 400 |
|  |  | 100-125 | 25 | 100 | 200 | 100 | 25 | 200 |
|  |  | 100-135 | 100 | 200 | 400 | 200 | 200 | 400 |
|  |  | 100-137 | 50 | 50 | 50 | 50 | 50 | 100 |
|  |  | **GMT** | **34** | **126** | **171** | **171** | **46** | **317** |
|  |  | **Mean** | **39** | **256** | **303** | **302** | **57** | **477** |
|  |  | **Median** | **25** | **100** | **200** | **200** | **50** | **200** |
|  | Typhim Vi  25 µg | 100-093 | 25 | 25 | 25 | 25 | 50 | 50 |
|  |  | 100-119 | 25 | 3200 | 3200 | 3200 | 25 | 6400 |
|  |  | 100-134 | 25 | 200 | 200 | 100 | 25 | 100 |
|  |  | **GMT** | **25** | **252** | **252** | **200** | **31** | **317** |
|  |  | **Mean** | **25** | **1142** | **1142** | **1108** | **33** | **2183** |
|  |  | **Median** | **25** | **200** | **200** | **100** | **25** | **100** |
|  | Placebo | 100-098 | 25 | 25 | 25 | 25 | 25 | 25 |
|  |  | 100-123 | 25 | 50 | 50 | 50 | 25 | 50 |
|  |  | 100-136 | 25 | 25 | 25 | 25 | 50 | 25 |
|  |  | **GMT** | **25** | **31** | **31** | **31** | **31** | **31** |
|  |  | **Mean** | **25** | **33** | **33** | **33** | **33** | **33** |
|  |  | **Median** | **25** | **25** | **25** | **25** | **25** | **25** |
